# Apolipoprotein E expression pattern in human induced pluripotent stem cells during *in vitro *neural induction

**DOI:** 10.12688/f1000research.23580.2

**Published:** 2020-08-24

**Authors:** Hyunah Lee, Paulina Nowosiad, Lucia M. Dutan Polit, Jack Price, Deepak P. Srivastava, Sandrine Thuret

**Affiliations:** 1 Department of Basic and Clinical Neuroscience, Institute of Psychiatry, Psychology, and Neuroscience, King's College London, London, SE5 9NU, UK

**Keywords:** Induced pluripotent stem cells, Neural stem cells, Directed differentiation, Apolipoprotein E

## Abstract

Apolipoprotein E (APOE) is a multifunctional protein that plays significant roles in important cellular mechanisms in peripheral tissues and is as well expressed in the central nervous system, notably by adult neural stem cells (NSCs) in the hippocampus. Evidence from animal studies suggest that APOE is critical for adult NSC maintenance. However, whether APOE has the potential to play a similar role in human NSCs has not been directly investigated. To address this question, we conducted a focused study characterising *APOE* gene and protein expression in an *in vitro* model of neural differentiation utilising human induced pluripotent stem cells. We found that *APOE* gene expression was dramatically decreased as the cells became more differentiated, indicating that *APOE* expression levels reflect the degree of cellular differentiation during neural induction. Furthermore, qualitative analysis results of immunocytochemistry showed that intracellular localisation of APOE protein becomes more pronounced as neural differentiation progresses. Taken together, our findings suggest a potential role for APOE in human NSC maintenance and justify further investigations being carried out to understand whether changes in APOE levels can directly impact the neurogenic capacity of human stem cells.

## Abbreviations

AD (Alzheimer’s disease); APOE (Apolipoprotein E); iPSCs (induced pluripotent stem cells); NSCs (neural stem cells)

## Introduction

Apolipoprotein E (APOE) is a pleiotropic protein that plays an important role in lipid metabolism ( Mahley & Rall, 2000) and is highly expressed in the brain mainly by glial cells ( Boyles *et al.*, 1985; Elshourbagy *et al.*, 1985). Although the primary function of APOE is lipid transport, its expression is also found in other cell types outside the context of lipid metabolism in the brain ( Liao *et al.*, 2017). For example, a recent single-cell RNA sequencing study on human post-mortem Alzheimer’s disease (AD) brains showed that activated microglia (relevant to the disease state) express high levels of APOE unlike naïve microglia (relevant to healthy/homeostatic state) in the prefrontal cortex, indicating that APOE expression is associated with immune function ( Mathys *et al.*, 2019). Furthermore, neuronal APOE can also be expressed at high levels under stress conditions such as brain injury although APOE expression is normally low in healthy neurons ( Mahley & Huang, 2012; Xu *et al.*, 2006). Interestingly, APOE is highly expressed in nestin/glial fibrillary acidic protein (GFAP) double-positive neural stem cells (NSCs) in the adult hippocampus of mice, and one of the phenotypes characterised in APOE-null mice is the premature depletion of NSC pool in the hippocampus, suggesting that NSC maintenance requires APOE expression ( Yang *et al.*, 2011).

Although the existing literature suggest that APOE plays an important role in stem cell maintenance, one should note that the majority of these findings were generated from rodent models. Since NSCs obtained from different species have been shown to behave in fundamentally different ways ( Mertens *et al.*, 2013; Otani *et al.*, 2016; Ray & Gage, 2006), characterisation of *APOE* expression in ‘human’ NSCs should be done prior to investigating its exact function. However, such evidence has not been reported to this date. To reduce this knowledge gap, we conducted a short study examining the expression pattern of APOE gene and protein in human induced pluripotent stem cells (iPSCs) undergoing neural induction *in vitro*. We found that gene expression is the highest in cells at the earliest stage of neural induction, whereas protein expression becomes more localised intracellularly, indicating that APOE expression pattern changes according to the differentiation state of cells.

## Methods

A list of materials used in this study is presented in Table 1.

**Table 1.** List of materials used in this study.

Name			Company	Catalogue Number
**CytoTune-iPS 2.0 Sendai** **Reprogramming Kit**			Thermo Fisher	A16517
**Essential 8™ medium**			Thermo Fisher	A1517001
**NUNC™ plates**			Thermo Fisher	140675
**Geltrex™**			Thermo Fisher	A1413302
**Versene (EDTA) solution**			Lonza	BE17-711E
**Hank's Balanced Salt Solution (HBSS)**			Thermo Fisher	14170-161
**Dulbecco’s Modified Eagle’s Medium/** **Nutrient Mixture F-12 Ham**			Sigma Aldrich	D6421
**GlutaMAX™**			Thermo Fisher	35050-061
**N-2 (100X)**			Thermo Fisher	17502-048
**Neurobasal® medium**			Thermo Fisher	21103-049
**B-27 minus vitamin A (50X)**			Thermo Fisher	12587-010
**LDN193189**			Sigma Aldrich	SML0559
**SB431542**			Sigma Aldrich	S4317
**XAV939**			Sigma Aldrich	X3004
**Cyclopamine**			LC Laboratories	C-8700
**Y-27632**			Sigma Aldrich	Y0503
**DNeasy Blood & Tissue Kit**			QIAGEN	69504
**Taq DNA Polymerase**			QIAGEN	201203
**HhaI digestion enzyme**			Thermo Fisher	ER1851
**TRIzol® reagent**			Thermo Fisher	15596026
**SuperScript® III First-Strand** **Synthesis System**			Thermo Fisher	18080051
**HOT FIREPol® EvaGreen® qPCR Mix**			Solis Biodyne	08-24-00001
Name	Concentration	Host/Clonality	Company	Catalogue Number
**Hoechst 33342**	1:2000	-	Thermo Fisher	H3570
**anti-human SOX2**	1:1000	Rabbit monoclonal	Millipore	AB5603
**anti-human TBR2**	1:250	Rabbit polyclonal	Abcam	AB23345
**anti-human ApoE**	1:200	Goat polyclonal	Millipore	AB947
**anti-Rabbit Alexa Fluor 488**	1:500	Donkey polyclonal	Thermo Fisher	A21206
**anti-Goat Alexa Fluor 594**	1:500	Donkey polyclonal	Thermo Fisher	A11058

### Cell line

CTR_M3_36S human induced pluripotent stem cell (iPSC) line was reprogrammed from keratinocytes obtained from a neurotypical male. Keratinocytes were reprogrammed by introducing a set of Sendai virus encoding human OCT4, SOX2, KLF4, and C-MYC (Yamanaka factors) using the CytoTune-iPS 2.0 Sendai Reprogramming Kit (Thermo Fisher) according to the manufacturer’s instructions. The virus was a gift from Dr. Mahito Nakanishi (AIST, Japan).

### Stem cell maintenance

Cells were regularly tested for mycoplasma and certified mycoplasma-free. iPSCs were maintained in Essential 8™ medium (Thermo Fisher) without antibiotics at 37°C, 5% CO _2_, 5% O _2_ in 6-well NUNC™ plates (Thermo Fisher) coated with Geltrex™ (Thermo Fisher). Passaging of iPSCs lines were done with Versene (EDTA) solution (Lonza) according to the manufacturer's instructions. Passaging ratio for iPSC maintenance was kept between 1:6 and 1:18.

### Directed differentiation

iPSC colonies approaching 80% confluence were passaged at 3:2 ratio on 6-well NUNC™ plates coated with Geltrex™ on D-2/-1 and maintained at 37°C, 5% CO _2_, 5% O _2_ for 24–48 hrs until they approached 100% confluence. Directed differentiation began on D0 by changing Essential 8™ medium to neural induction medium and incubating the cells at 37°C, 5% CO _2_, 20% O _2_. Neural induction lasted for 7 days. To prepare neural induction medium, N2:B27 was first prepared by mixing the N2 medium (Dulbecco’s Modified Eagle’s Medium/Nutrient Mixture F-12 Ham (DMEM/F12) (Sigma Aldrich) supplemented with 1X GlutaMAX™ (Thermo Fisher) and 1X N-2 supplement (Thermo Fisher)) and the B27 medium (Neurobasal® medium (Thermo Fisher) supplemented with 1X GlutaMAX™ and 1X B-27 supplement (Thermo Fisher) or 1X B-27 without vitamin A supplement (Thermo Fisher)) at 1:1 ratio. The following small molecule inhibitors were added to N2:B27 to make the neural induction medium: 100 nM LDN193189 (Sigma Aldrich) and 10 µM SB431542 (Sigma Aldrich) for dual SMAD inhibition (DSi); 100 nM LDN193189, 10 µM SB431542, and 2 µM XAV939 (Sigma Aldrich) for dual SMAD inhibition plus Wnt/β-catenin inhibition (DS-Wi); and 100 nM LDN193189, 10 µM SB431542, 2 µM XAV939, and 1 µM Cyclopamine (LC Laboratories) for dual SMAD inhibition plus Wnt/β-catenin plus sonic hedgehog inhibition (DS-WHi). Neural induction medium was used from D0 to D7, and N2:B27 was used from D8 onwards. Medium was changed every 24 hrs throughout the entire directed differentiation period.

Neural passaging 1, 2, and 3 were performed with Accutase (Thermo Fisher) on D7, D12, and D15/16, respectively. Briefly, cells were washed with room temperature HBSS and treated with Accutase at 37°C, 5% CO _2_, 5% O _2_ for 3–4 minutes. Cold Accutase was used for neural passagings 1 and 2, and room temperature Accutase was used for neural passaging 3. Cells in Accutase were then collected with a P1000 pipette. Extra care was taken during neural passagings 1 and 2 where P1000 pipetting was done no more than 5 times when cells in Accutase were collected. Collected cells were then mixed with room temperature DMEM/F12 (twice the volume of Accutase used) so that Accutase could be deactivated, and centrifugation was performed twice to wash off the Accutase from cells. Centrifugation was done at 900 revolutions per minute (RPM) for 2 min during neural passaging 1 and 2, and at 1250 RPM for 2 min during neural passaging 3. After centrifugation, cells were plated on new 6-well NUNC™ plates coated with Geltrex™. Passaging ratios were 1:1 for neural passaging 1 and 2, and 2:3 for neural passaging 3. To ensure cell survival 10 µM Y-27632 (Sigma Aldrich), a Rho-associated coiled-coil containing protein kinase (ROCK) inhibitor, was mixed with the plating medium at each neural passaging and then removed after 24 hrs.

### Genotyping

Genomic DNA was extracted from iPSCs using the DNeasy Blood & Tissue Kit (QIAGEN) according to the manufacturer’s instructions. The *APOE* locus containing the rs429358 and rs7412 SNPs was amplified with Taq DNA Polymerase (QIAGEN) according to the manufacturer's instructions. Briefly, the reaction mix containing 1X PCR Buffer, 1X Q-Solution, 10 mM dNTP mix (0.2 mM final concentration), primers (forward and reverse each at 0.4 µM final concentration), 1.25 units Taq polymerase, and 1 µg of genomic DNA was incubated at 95°C for 4 mins to activate the Taq polymerase. Then, 35 cycles of ‘denaturation at 94°C for 30 secs, annealing at 68°C for 30 secs, elongation at 72°C for 1 min’ was performed. Then, final extension was done at 72°C for 10 mins. This polymerase chain reaction (PCR) was done with S1000 Thermal Cycler (Bio-Rad). The primers used for *APOE* genotyping ( Table 2) were previously designed by Henderson and colleagues ( Henderson *et al.*, 2002), and they are able to generate PCR products that can be visualised easily by gel electrophoresis after HhaI enzyme (Thermo Fisher) digestion. Amplified PCR products were digested with 1 unit of HhaI digestion enzyme and gel electrophoresis was performed using a 3% agarose gel containing 0.5 µg/mL ethidium bromide. Raw dataset is available as an **underlying data** via Figshare (doi: 10.6084/m9.figshare.12199721.v1).

**Table 2.** Sequence of primers used in this study.

Genotyping	Forward	Reverse
***APOE***	GAC GCG GGC ACG GCT GTC CAA GGA GCT GCA GGC GAC GCA GGC CCG GCT GGA CGC GGA CAT GGA GGA	AGG CCA CGC TCG ACG CCC TCG CGG GCC CCG GCC TGG TAC ACT
Gene expression	Forward	Reverse
***APOE***	GTT GCT GGT CAC ATT CCT GG	GCA GGT AAT CCC AAA AGC GAC
***GAPDH***	AGC CTC AAG ATC ATC AGC AA	CTG TGG TCA TGA GTC CTT CC

### Gene expression analysis

Total RNA was extracted from D7, D12, D15/16, and D18/19 cells that were not used for neural passaging with TRIzol® reagent (Thermo Fisher) according to manufacturer’s instructions and eluted in 25–30 µL of diethyl pyrocarbonate (DEPC)-treated water. Reverse transcription of total RNA into complementary DNA was performed using SuperScript® III First-Strand Synthesis System (Thermo Fisher) according to the manufacturer's instructions. Briefly, the random hexamers were annealed to total RNA at 25°C for 10 mins, then the synthesis was performed at 50°C for 50 mins, and then the reaction was terminated at 85°C for 5 mins. The final product was diluted to 5 ng/µL of total RNA converted to cDNA using DEPC-treated water. S1000 Thermal Cycler (Bio-Rad) was used for reverse transcription.

For gene expression analysis, real-time quantitative polymerase chain reaction (qPCR) was performed using the HOT FIREPol® EvaGreen® qPCR Mix (Solis Biodyne) according to the manufacturer’s instructions. Briefly, the reaction mix containing the HOT FIREPol® EvaGreen® qPCR Mix, primers (forward and reverse each at 0.2 µM final concentration), and cDNA was incubated at 95°C for 15 mins to activate the HOT FIREPol® DNA polymerase, then 45 cycles of ‘denaturation at 95°C for 30 secs, annealing at 60°C for 30 secs, elongation at 72°C for 30 secs’ was performed. Melting curve analysis was done on each gene based on the melting profile generated every 1°C increment between 60°C and 95°C. MJ Research PTC-200 Thermal Cycler (Bio-Rad) was used for qPCR. The sequence of primers are presented in Table 2. C _T_ values of *APOE* were normalised to that of *GAPDH*, and relative expression of *APOE* across samples were quantified using the 2 ^-ΔΔCt^ method where D7 sample was used as a reference for each differentiation lineage. Raw dataset is available as an **underlying data** via Figshare (doi: 10.6084/m9.figshare.12136944).

### Immunocytochemistry

Cells were fixed with 4% paraformaldehyde for 10 mins at room temperature, permeabilized with 0.1% Triton™ X-100 in 1X Tris-buffered saline (TBS) for 15–30 minutes, and then blocked with 5% normal donkey serum in TBS for 30 minutes. Primary antibodies were incubated at 4°C overnight followed by 3 washings with TBS. Secondary antibodies conjugated with fluorescent dyes were incubated at room temperature for 1 hours followed by 2 washings with TBS. Nuclei were stained with 5 µg/mL Hoechst® 33342 solution (Thermo Fisher) for 30 seconds immediately prior to imaging, and cells were washed with TBS 2 times after nuclear staining. All primary antibodies were diluted in 5% normal donkey serum in TBS, secondary antibodies in 1% normal donkey serum in TBS, and Hoechst® 33342 solution in TBS. Imaging was done with IX 70 inverted epifluorescence microscope (Olympus) connected to AxioVision imaging software (version 4.4). Scale bars were inserted on the images using ImageJ software (version 1.49c). CellProfiler (version 3.1.9) was used to quantify the percentage of cells immunopositive for SOX2, TBR2, and APOE at the intracellular regions. Raw dataset for the images is available as an **underlying data** via Figshare (doi: 10.6084/m9.figshare.12199745.v1). Raw dataset for the quantification is available as an **underlying data** via Figshare (doi: 10.6084/m9.figshare.12781604.v1).

### Statistical analysis

GraphPad Prism (version 8.4.2.679) was used for statistical analysis. The statistical significance of the mean differences between groups were analysed by one-way analysis of variance (ANOVA) followed by Bonferroni correction for multiple testing. The mean, standard error of measurement (SEM), and number of biological replicates are reported. P-value < 0.05 was considered significant to reject the null hypothesis that the differences observed between groups is due to random variation.

## Results

**Figure 1.**
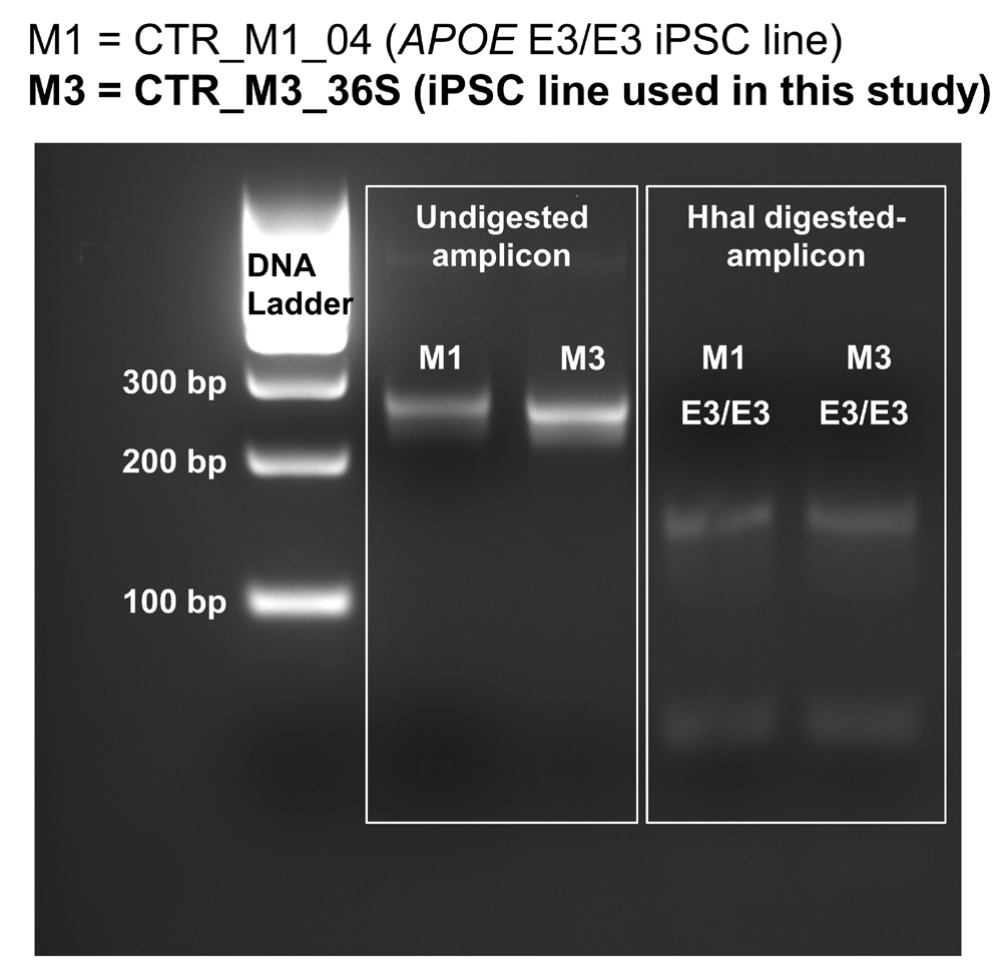
*APOE* genotyping of the cell line used in this study. CTR_M3_36S human iPSC line derived from a neurotypical male is homozygous for *APOE3* (denoted as M3 in this figure). CTR_M1_04 human iPSC line that was known to be homozygous for *APOE3* was used as control (denoted as M1 in this figure). HhaI-digested PCR amplicons were run on a 3% agarose gel, and the band loci were compared with the data previously reported by Henderson and colleagues who developed this genotyping method ( Henderson *et al*., 2002). The band loci for both CTR_M3_36S and CTR_M1_04 lines match with the homozygous *APOE3* data reported by Henderson and colleagues (see Figure 1 of Henderson *et al*., 2002).

**Figure 2.**
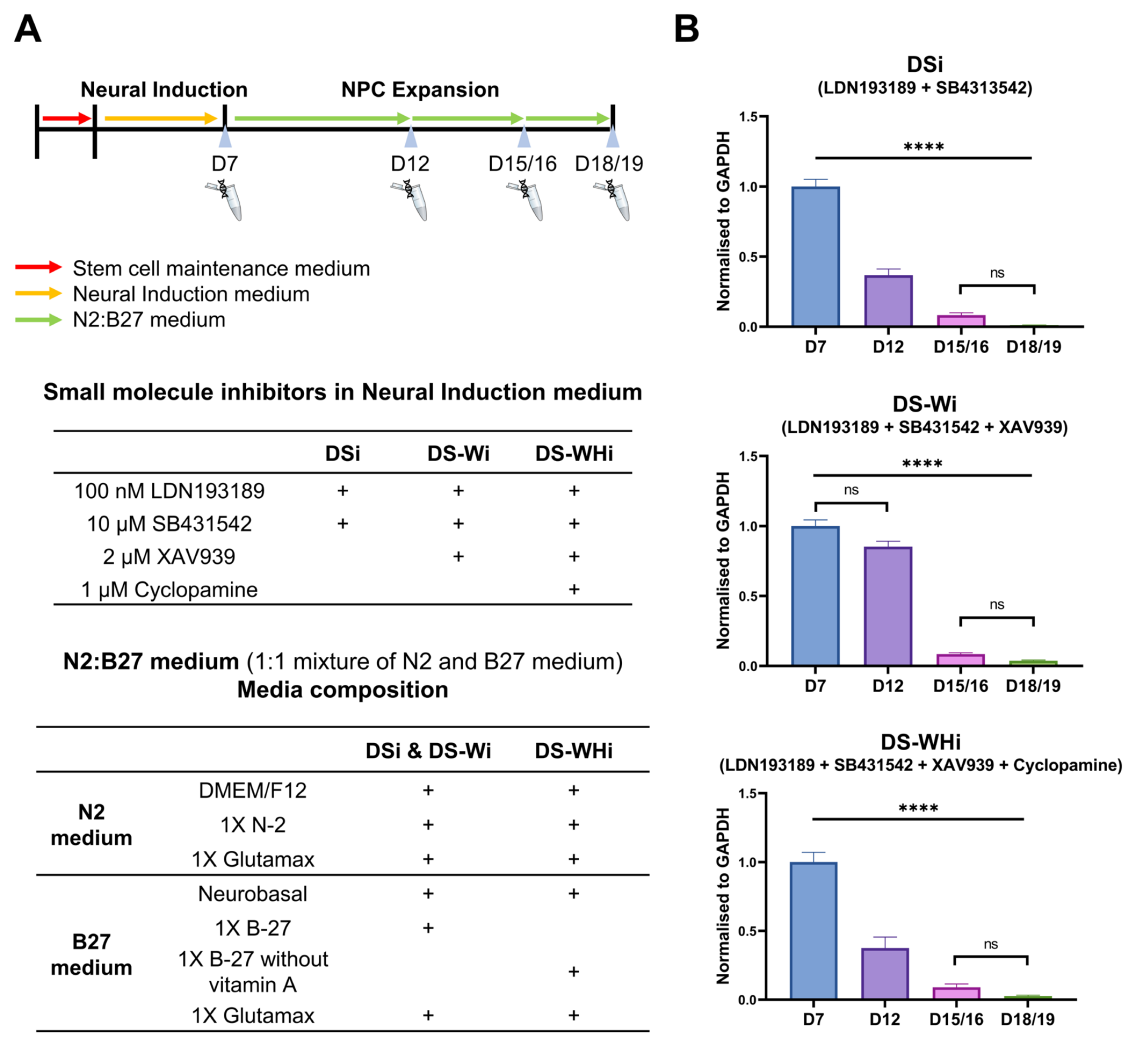
*APOE* gene expression changes according to the differentiation state of cells during *in vitro* directed differentiation. **A**) Schematic diagram of directed differentiation. CTR_M3_36S iPSCs were maintained in stem cell maintenance medium after replating (D-2/-1). On D0 neural induction began by changing the stem cell maintenance medium to neural induction medium. N2:B27 was used from D8 onwards. Medium was changed every 24 hrs throughout the entire differentiation period. Neural passaging 1, 2, and 3 were carried out on D7, D12, and D15/16, respectively. Total RNA extraction was made on cells that were not used for neural passaging on D7, D12, D15/16, and D18/19. Neural induction medium composition for each differentiation lineage and N2:B27 medium composition are also shown. **B**) *APOE* gene expression is reduced along neural induction regardless of lineage. Real-time qPCR was performed on CTR_M3_36S iPSCs undergoing directed differentiation at D7, D12, D15/16, and D18/19. *APOE* expression was normalised to that of *GAPDH*. D7 samples were used as reference samples for each lineage. One-way ANOVA with Bonferroni correction. n = 3. Mean (bars) with S.E.M. (error bars) shown. **** ANOVA p-value < 0.0001. ns: non-significant after Bonferroni correction. DSi: dual SMAD inhibition. DS-Wi: DSi plus Wnt/β-catenin inhibition. DS-WHi: DS-Wi plus sonic hedgehog inhibition.

To characterise the expression of APOE in human stem cells undergoing neural induction, an iPSC line derived from a neurotypical male with *APOE3* homozygous genotype (CTR_M3_36S cell line) ( Figure 1; ( Lee, 2020b)) ( Deans *et al.*, 2017) were differentiated into neural lineages. Genotyping of CTR_M3_36S was performed using the method developed by Henderson and colleagues ( Henderson *et al.*, 2002), and CTR_M3_36S was confirmed to be homozygous for *APOE3* by comparing the data with that of an *APOE3* homozygous cell line (CTR_M1_04) that reported by Henderson and colleagues ( Henderson *et al.*, 2002, Figure 1). Neural induction into dorsal forebrain progenitors was performed using modified dual SMAD inhibition protocols ( Cocks *et al.*, 2014; Deans *et al.*, 2017; Kathuria *et al.*, 2018; Yu *et al.*, 2014), where combinations of small molecule inhibitors were used to inhibit bone morphogenetic protein (BMP), transforming growth factor (TGF)-β, Wnt/β-catenin, and sonic hedgehog signalling pathways from D0 to D7 of neural induction ( Figure 2A).

**Figure 3.**
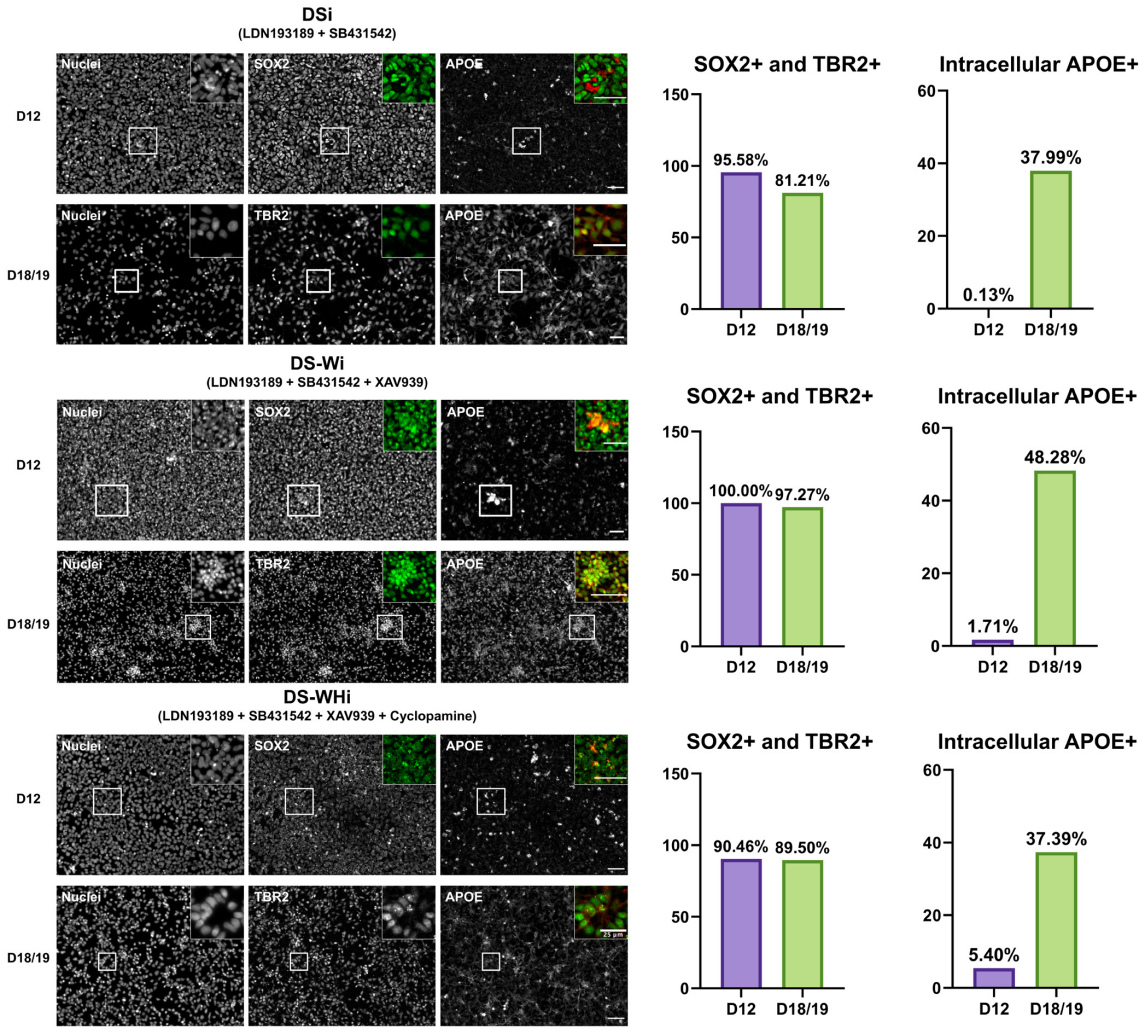
APOE localisation pattern becomes more intracellular with *in vitro* directed differentiation. APOE protein is more localised intracellularly in differentiated cells. (Left) Representative images of cells at D12 and D18/19 expressing SOX2 (NSC marker) and TBR2 (NPC marker), respectively. Insets show images of SOX2/TBR2 in green and APOE in red. Scale bars indicate 50 µm unless stated otherwise. (Right) Quantification of SOX2-/TBR2-positive cells and intracellular APOE-positive cells amongst SOX2-TBR2-positive cells. DSi: dual SMAD inhibition. DS-Wi: DSi plus Wnt/β-catenin inhibition. DS-WHi: DS-Wi plus sonic hedgehog inhibition.

Gene expression analysis revealed that *APOE* expression was the highest at D7, and the drastic down-regulation of *APOE* from D7 > to D18/19 was observed regardless of the combination of small molecule inhibitors used from D0 to D7 (p < 0.0001) ( Figure 2B, **underlying data** ( Lee, 2020a)). Immunocytochemistry showed that D12 and D18/19 cells expressed SRY-box transcription factor 2 (SOX2), a NSC marker, and T-Box Brain Protein 2 (TBR2), a neural progenitor cell (NPC) marker, respectively, for all combinations of small molecule inhibitors used from D0 to D7. Qualitative analysis of immunocytochemistry results revealed that ApoE became more localised to the intracellular region at D18/19 compared to D12 ( Figure 3; ( Lee, 2020c; Lee, 2020d)).

## Discussion

Unlike the existing animal models of *APOE* deficiency and humanised *APOE* expression where genetic modifications were introduced globally (whole body) rather than specifically to NSCs, the *in vitro* model used in this study allowed us to examine *APOE* expression pattern exclusively in stem cells that were pushed towards the neural lineage. Our findings demonstrate that in cells at the earliest stage of neurodevelopment, 1) human *APOE* gene expression is high, and 2) APOE protein is not clearly localised at the intracellular region. Various combinations of small molecule inhibitors did not alter these patterns of expression.

Although further investigations will be needed to understand the exact role of APOE in neurodevelopment, the existing literature seems to suggest that APOE can be both downstream and upstream of stem cell maintenance. For example, several chromatin precipitation studies have shown that POU class 5 homeobox 1 (POU5F1), SOX2, Kruppel like factor 4 (KLF4), MYC proto-oncogene (MYC) and Nanog Homeobox (NANOG) all bind to the promoter region of *APOE*, suggesting that *APOE* expression could be directly regulated by such stem cell maintenance factors ( ENCODE Project Consortium, 2004; ENCODE Project Consortium, 2011; Kim *et al.*, 2008; Lachmann *et al.*, 2010; Liu *et al.*, 2008; Marson *et al.*, 2008). However, other evidence suggests that APOE itself could be a direct regulator of cell fate determination. Meyer and colleagues ( Meyer *et al.*, 2019) showed that changing the *APOE* genotype from ε4 (AD risk factor) to ε3 (neutral genotype) in human NPCs can suppress premature neuronal differentiation and maturation via increasing the transcription repressor activity of RE1 silencing transcription factor (REST). Interestingly, *APOE* mRNA levels were lower in ε4 NPCs compared to ε3 NPCs, suggesting higher *APOE* gene expression is indeed likely to be associated with the undifferentiated state of NPCs. As a follow-up to our findings and the existing literature, we propose that further investigations should be carried out to elucidate the role of APOE in stem cell maintenance. For example, one could examine whether prolonged expression and/or overexpression of *APOE* gene in human NPCs can suppress further differentiation in these cells.

In this study, qualitative analysis was performed on APOE immunocytochemistry results. As the cells became more differentiated from NSCs to NPCs, APOE localisation pattern became more clearly intracellular. To validate this observation, co-localisation analysis with cytoskeletal proteins (such as Tubulin beta-3 chain and Microtubule-associated protein 2) or with plasma membrane proteins (such as N-Cadherin) will be needed in future investigations. Furthermore, APOE has been shown to exist in both secreted and intracellular forms in the existing literature ( Huang & Mahley, 2014). Therefore, it will also be important to examine which form of APOE is produced at each differentiation stage. It is possible that more APOE is secreted in undifferentiated cells compared to differentiated cells, which may not be fully captured using immunocytochemistry techniques performed on fixed cells. Interestingly, Gan and colleagues previously reported that APOE is indeed secreted by NSCs as well as NPCs, and secreted APOE was found to play a vital role in regulating NSC survival and neurosphere formation ( Gan *et al.*, 2011). Further investigations on changes of secreted and intracellular protein levels throughout neural differentiation will be able to clarify whether cells indeed produce different forms and levels of APOE depending on its differentiation state. This will in turn provide more definitive clues to whether APOE plays a stage-dependent role in neurodevelopment.

In this study, qualitative analysis was performed on APOE immunocytochemistry results. As the cells became more differentiated from NSCs to NPCs, APOE localisation pattern became more intracellular. To validate this observation, however, additional experiments with a more direct quantitative approach should be conducted. For example, APOE protein levels in various subcellular compartments could be measured and compared by performing Western Blot. Since APOE has been shown to exist in both secreted and intracellular forms ( Huang & Mahley, 2014), it will be interesting to see which form of APOE is produced at each differentiation stage. It is possible that more APOE is secreted in undifferentiated cells compared to differentiated cells, which may not be fully captured using immunocytochemistry techniques performed on fixed cells. Interestingly, Gan and colleagues previously reported that APOE is indeed secreted by NSCs as well as NPCs, and secreted APOE was found to play a vital role in regulating NSC survival and neurosphere formation ( Gan *et al.*, 2011). Therefore, further investigations on secreted and intracellular APOE using quantitative approaches will be able to clarify whether cells indeed produce different forms and levels of APOE depending on its differentiation state. This will in turn provide more definitive clues to whether APOE plays a stage-dependent role in neurodevelopment.

One limitation of this study is that the time-dependent changes of differentiation markers such as SOX2 and TBR2 were not examined alongside APOE. It is worth noting, however, that TBR2 was shown to be capable of suppressing SOX2 expression during differentiation of NSCs to NPCs ( Hodge *et al.*, 2012). Given this information, it is unlikely that TBR2-positive cells observed in this study at D18/19 will simultaneously express high levels of SOX2. However, time-dependent changes of various markers of differentiation would add further validity to our observations and unequivocally clarify whether APOE expression is indeed correlated with the differentiation state of the cells. Another limitation of this study is that the exact locus of APOE expression could not be examined in detail using a standard epifluorescence microscope in this study. High-resolution microscopy techniques (such as confocal microscopy) would have been more ideal to identify the accurate loci of APOE expression and overcome the challenges of imaging densely packed cells at the earliest stages of neural induction (D0–D7). Further investigations with improved imaging capacity will therefore allow us to characterise APOE during the earlier stages of neural induction and hint at potential mechanisms underlying its role in neurodevelopment.

Since NSCs derived from iPSCs *in vitro* may not fully resemble the developmental and postnatal NSCs found *in vivo*, APOE expression should be further investigated in animal models of brain development as well. The most direct evidence of *in vivo* APOE expression in NSCs to this date comes from a study by Yang and colleagues, where Nestin-positive NSCs in the mouse developing dentate gyrus was isolated using fluorescence-activated cell sorting, and APOE expression was examined from as early as postnatal day 7 (P7) ( Yang *et al.*, 2011). NSCs at P7 had low expression of APOE which increased with the age of mice, and the deletion of APOE had detrimental effects on the maintenance of stem cells in the dentate gyrus. Although these findings clearly demonstrate the importance of APOE in brain development, the study had limitations in that prenatal NSCs were not examined, and functional studies of APOE were based on global rather than conditional knockouts. Furthermore, Yang and colleagues’ data cannot be directly compared with our dataset due to species difference and the lack of detailed characterisation of NSCs in this study. To address this knowledge gap, more data from both *in vitro* and *in vivo* samples derived from various species should be generated and compared against each other. We hope that our focused study has laid a strong foundation to such collaborative investigations that may be conducted in the future.

In conclusion, we report that human *APOE* gene expression levels are highly correlated with the undifferentiated state of cells during directed differentiation *in vitro*, and ApoE protein is localised more in the intracellular region in cells at later stages of differentiation. Combining our observations and previous evidence reported in the literature, we speculate that APOE has an important role in stem cell maintenance and propose that further investigations should be carried out to validate our findings including methods that were not employed in this study. Moreover, it would be interesting to examine the exact underlying mechanisms such as 1) whether APOE is an upstream or downstream factor of stem cell maintenance, and 2) whether *APOE4* genotype and APOE loss-of-function would produce similar phenotypes.

## Data availability

### Underlying data

Figshare: raw data for qPCR. https://doi.org/10.6084/m9.figshare.12136944.v1 ( Lee, 2020a)

This project contains the following underlying data:

- Lee *et al.* raw data for qPCR.csv (C(t) values, efficiency of amplification, and values calculated for normalised gene expression analysis for APOE.)

Figshare: raw data for Genotyping. https://doi.org/10.6084/m9.figshare.12199721.v1 ( Lee, 2020b)

This project contains the following underlying data:

- Lee *et al.* raw data for Genotyping.TIF (Gel image used in Figure 1. Genomic DNA from human iPSCs amplified for the APOE locus, then digested with HhaI enzyme. Run on 3% agarose gel.)

Figshare: raw data for D12 and D18/19 immunocytochemistry. https://doi.org/10.6084/m9.figshare.12199745.v1 ( Lee, 2020c)

This project contains the following underlying data:

- 01 Lee *et al.* raw data for DS D12 Hoechst.tif
- 02 Lee *et al.* raw data for DS D12 SOX2.tif
- 03 Lee *et al.* raw data for DS D12 APOE.tif
- 04 Lee *et al.* raw data for DS D18-19 Hoechst.tif
- 05 Lee *et al.* raw data for DS D18-19 TBR2.tif
- 06 Lee *et al.* raw data for DS D18-19 APOE.tif
- 07 Lee *et al.* raw data for DS+Wi D12 Hoechst.tif
- 08 Lee *et al.* raw data for DS+Wi D12 SOX2.tif
- 09 Lee *et al.* raw data for DS+Wi D12 APOE.tif
- 10 Lee *et al.* raw data for DS+Wi D18-19 Hoechst.tif
- 11 Lee *et al.* raw data for DS+Wi D18-19 TBR2.tif
- 12 Lee *et al.* raw data for DS+Wi D18-19 APOE.tif
- 13 Lee *et al.* raw data for DS+WHi D12 Hoechst.tif
- 14 Lee *et al.* raw data for DS+WHi D12 SOX2.tif
- 15 Lee *et al.* raw data for DS+WHi D12 APOE.tif
- 16 Lee *et al.* raw data for DS+WHi D18-19 Hoechst.tif
- 17 Lee *et al.* raw data for DS+WHi D18-19 TBR2.tif
- 18 Lee *et al.* raw data for DS+WHi D18-19 APOE.tif
(Images were taken with IX70 inverted epifluorescence microscope (Olympus) connected to AxioVision imaging software 4.4. ImageJ 1.49c was used to generate TIFF files.)

Figshare: raw data for quantification of D12 and D18/19 immunocytochemistry. https://doi.org/10.6084/m9.figshare.12781064.v1 ( Lee, 2020d)

This project contains the following underlying data:

- pipeline_01_DSi.cppipe
- pipeline_02_DS-Wi.cppipe
- pipeline_03_DS-WHi.cppipe
- quantification.xlsx
(cppipe files: CellProfiler v.3.1.9 was used to generate the pipelines for each image set (one differentiation protocol, 3 channels, 2 timepoints. quantification.xlsx: The raw data generated after running the pipelines were copied to a spreadsheet. 'Cells / Nuclei (%)' column shows the percentage of cells positive for SOX2 at D12 and TBR2 at D18/19. 'Intracellular APOE / Cells (%)' column shows the percentage of intracellular APOE-positive cells amongst SOX2- and TBR2-positive cells. These 2 columns are presented as bar graphs on Figure 3 of the manuscript.)

Data are available under the terms of the Creative Commons Attribution 4.0 International license (CC-BY 4.0).
