# Supplementary material for: Apolipoprotein E expression pattern in human induced pluripotent stem cells during in vitro neural induction
Source: F1000Res. 2020 Aug 24;9:353. Originally published 2020 May 12. [Version 2] doi: 10.12688/f1000research.23580.2 (PMC7355222; doi:10.12688/f1000research.23580.2)
Supplement: Peer review history

# Peer review: Reviewer response for version 2

**DOI:** 10.5256/f1000research.28981.r70146

**Published:** 2022-12-15

**Authors:** Jade Marsh

**Affiliations:** 1 University of Oxford, Oxford, UK; 2 Kavli Institute for Nanoscience Discovery, Oxford, UK

The authors have addressed my concerns to a satisfactory level.

Is the work clearly and accurately presented and does it cite the current literature?

Yes

If applicable, is the statistical analysis and its interpretation appropriate?

Yes

Are all the source data underlying the results available to ensure full reproducibility?

Yes

Is the study design appropriate and is the work technically sound?

Yes

Are the conclusions drawn adequately supported by the results?

Partly

Are sufficient details of methods and analysis provided to allow replication by others?

Yes

Reviewer Expertise:

Molecular and cellular neuroscience

I confirm that I have read this submission and believe that I have an appropriate level of expertise to confirm that it is of an acceptable scientific standard.

# Peer review: Reviewer response for version 2

**DOI:** 10.5256/f1000research.28981.r70145

**Published:** 2020-09-07

**Authors:** Noelia Urbán

**Affiliations:** 1 Institute of Molecular Biotechnology of the Austrian Academy of Sciences (IMBA), Vienna Biocenter Campus (VBC), Vienna, Austria

The authors have either addressed my concerns or clearly discussed the limitations of the study. I have no further comments.

Is the work clearly and accurately presented and does it cite the current literature?

Yes

If applicable, is the statistical analysis and its interpretation appropriate?

Yes

Are all the source data underlying the results available to ensure full reproducibility?

Yes

Is the study design appropriate and is the work technically sound?

Yes

Are the conclusions drawn adequately supported by the results?

Partly

Are sufficient details of methods and analysis provided to allow replication by others?

Yes

Reviewer Expertise:

Adult neurogenesis

I confirm that I have read this submission and believe that I have an appropriate level of expertise to confirm that it is of an acceptable scientific standard.

# Peer review: Reviewer response for version 1

**DOI:** 10.5256/f1000research.26021.r65479

**Published:** 2020-07-10

**Authors:** Jade Marsh

**Affiliations:** 1 University of Oxford, Oxford, UK; 2 Kavli Institute for Nanoscience Discovery, Oxford, UK

**Summary:**

- Previous studies in mice have shown that APOE is highly expressed by neural stem cells (NSCs) and that it has an isoform-dependent regulatory role in their maintenance. Whether this function extends to human NSCs is not known. Here, the authors begin to address this knowledge gap by characterising the expression of APOE in APOE3/APOE3 human induced pluripotent stem cell (iPSC)-derived NSCs undergoing differentiation. Through qPCR and immunocytochemistry (ICC) techniques, the authors show a reduction in APOE gene expression during the differentiation of NSCs to NPCs and observe a change in the cellular distribution of APOE.

 

**General comments:**

- This short study is concise and provides preliminary but important data to contribute to our understanding of the role of APOE in NSCs of human origin. Although the qPCR data is robust and clearly demonstrates a significant decrease in APOE gene expression as NSCs differentiate, the qualitative assessment of the ICC data is not as convincing.

 

**Specific comments:**

- Fig 2 C: APOE appears to be more widely expressed at D18/19 for all three NSC lineages (DSi, DS-Wi and DS-WHi). Quantification of the signal intensities of APOE and the differentiation markers should be carried out to validate the conclusion that APOE expression decreases as NSCs differentiate.
- Fig 2 C: It may be more helpful and striking to compare D18/19 images with D7 images instead of D12 since APOE mRNA levels are highest at this time point. Notably, D7 is also used as the baseline for the qPCR data.
- The authors describe an increase in intracellular localisation of APOE following NSC differentiation – providing higher magnification images may reveal changes in APOE distribution more clearly.

Is the work clearly and accurately presented and does it cite the current literature?

Yes

If applicable, is the statistical analysis and its interpretation appropriate?

Yes

Are all the source data underlying the results available to ensure full reproducibility?

Yes

Is the study design appropriate and is the work technically sound?

Yes

Are the conclusions drawn adequately supported by the results?

Partly

Are sufficient details of methods and analysis provided to allow replication by others?

Yes

Reviewer Expertise:

Molecular and cellular neuroscience

I confirm that I have read this submission and believe that I have an appropriate level of expertise to confirm that it is of an acceptable scientific standard, however I have significant reservations, as outlined above.

# Response

**Published:** 2020-08-19

**Authors:** Sandrine Thuret

**Affiliations:** King's College London, UK

*Comment 1. Fig 2 C: APOE appears to be more widely expressed at D18/19 for all three NSC lineages (DSi, DS-Wi and DS-WHi). Quantification of the signal intensities of APOE and the differentiation markers should be carried out to validate the conclusion that APOE expression decreases as NSCs differentiate.*

The authors would like to thank the reviewer for the comment on the quantification of ICC images. We now include a quantification of the images in the updated manuscript. The percentage of SOX2+ cells and TBR2+ cells at D12 and D18/19, as well as the percentage of SOX2+ and TBR2+ cells that showed immunostaining patterns for intracellular APOE were quantified using CellProfiler v3.1.9. **The quantified data presented as bar graphs can be found in Figure 3; the analysis procedure can be found in the ‘Methods (Immunocytochemistry)’ section; and the analysis pipeline as well as the raw data for quantification can be found on Figshare which can be accessed by the following DOI:** https://doi.org/10.6084/m9.figshare.12781064.v1 **.** The results indicate a higher percentage of intracellular APOE/TBR2+ cells at D18/19 compared to intracellular APOE/SOX2+ cells at D12, supporting our conclusions made in the manuscript prior to the inclusion of these quantification data.

*Comment 2. Fig 2 C: It may be more helpful and striking to compare D18/19 images with D7 images instead of D12 since APOE mRNA levels are highest at this time point. Notably, D7 is also used as the baseline for the qPCR data.*

The authors would like to thank the reviewer for mentioning this important aspect of the ICC experiment reported in our manuscript. **While the authors confirm that the ICC experiments were conducted for APOE on D7 cells, the data were not included in the manuscript due to the following reasons. **According to the differentiation protocol, the cells were maintained at high density approaching near 100% confluence from D0 to D7. We observed that this inadvertently diminishes the quality of immunocytochemistry images for D7 cells, since clear boundaries of nuclei could not be easily identified with epifluorescence microscopy and further complicated the downstream quantification process. The possibility of dissociating D7 cells and plating them on to a different surface for better image quality and quantification was considered briefly. However, such additional handling was not done to the cells so that any potential source of artefacts that could mask the true state of D7 cells can be ruled out in our experiments. While the use of epifluorescence microscopy in our study can be seen as a clear limitation, APOE immunostaining patterns of D7 cells was not qualitatively different from that of D12 cells in our observations. Further investigations using three-dimensional imaging techniques such as confocal microscopy will enable better imaging and quantification of densely packed cells on D7. **The ‘Discussion’ section in the updated manuscript now includes a new paragraph regarding this aspect.**

“One limitation of this study is that the time-dependent changes of differentiation markers such as SOX2 and TBR2 were not examined alongside APOE. It is worth noting, however, that TBR2 was shown to be capable of suppressing SOX2 expression during differentiation of NSCs to NPCs ( Hodge et al., 2012). Given this information, it is unlikely that TBR2-positive cells observed in this study at D18/19 will simultaneously express high levels of SOX2. However, time-dependent changes of various markers of differentiation would add further validity to our observations and unequivocally clarify whether APOE expression is indeed correlated with the differentiation state of the cells. **Another limitation of this study is that the exact locus of APOE expression could not be examined in detail using a standard epifluorescence microscope in this study. High-resolution microscopy techniques (such as confocal microscopy) would have been more ideal to identify the accurate loci of APOE expression and overcome the challenges of imaging densely packed cells at the earliest stages of neural induction (D0-D7). Further investigations with improved imaging capacity will therefore allow us to characterise APOE during the earlier stages of neural induction and hint at potential mechanisms underlying its role in neurodevelopment.**”

*Comment 3. The authors describe an increase in intracellular localisation of APOE following NSC differentiation – providing higher magnification images may reveal changes in APOE distribution more clearly.*

The authors would like to thank the reviewer for this comment. We agree that the resolution of the images in Figure 2 is not as high as the original data when the manuscript is viewed/downloaded online. **In the updated manuscript, these images are shown in a separate figure (Figure 3).** The PDF version should enable sufficient magnification to view the composite panels and clearly demonstrate more intracellular localisation on D18/19 cells. **In addition, as indicated in the ‘Data availability’ section of the submitted manuscript, the original data with higher resolutions for all images can be found on Figshare, a public repository for scientific data, which can be accessed via the following DOI:** https://doi.org/10.6084/m9.figshare.12199745.v1 **.** We are confident that the patterns of APOE immunocytochemistry (ICC) reported in the manuscript can be confirmed with greater detail from the raw data available on Figshare.

# Peer review: Reviewer response for version 1

**DOI:** 10.5256/f1000research.26021.r65480

**Published:** 2020-07-06

**Authors:** Noelia Urbán

**Affiliations:** 1 Institute of Molecular Biotechnology of the Austrian Academy of Sciences (IMBA), Vienna Biocenter Campus (VBC), Vienna, Austria

Summary:

 APOE is highly expressed by neural stem cells (NSCs) in the hippocampus of adult mice, particularly in the quiescent state, and has been associated with stem cell maintenance. However, no data is available on the expression pattern of APOE in human neural stem cells. The authors monitor APOE expression by QPCR and immunocytochemistry during the neuronal differentiation of iPSCs using three different types of neural induction media. They report a dramatic reduction in APOE mRNA levels during differentiation, as well as a change in the cellular distribution of APOE protein.

Comments:

 Although preliminary, the changes in APOE expression are an interesting and important observation. However, while the QPCR data is convincing and very robust, the immunocytochemistry studies should be further analysed/improved in order to draw any strong conclusions. The images presented are not of very good quality, and if judging by them, APOE expression rather seems to increase globally during differentiation, with few cells expressing high levels at D12 and most cells expressing moderate levels at D18/19. My main specific recommendations are:

- If possible, provide higher magnification/higher quality images of APOE stainings, including also the other time points during differentiation. Day 7 would be particularly important to include, since it displays the highest levels of expression by QPCR.
- APOE stainings should be quantified to support the conclusions that its expression decreases during differentiation and there is a change in the protein localization. If this is not possible, the conclusions should be toned down and further experiments suggested in the discussion (for example, protein quantification by WB and cellular fractionation and quantification of protein in the media to assess intracellular protein localization and secretion, respectively).
- In the discussion, it should be noted that iPSC-derived NSCs might not fully resemble adult NSCs. A brief discussion of what is known about the expression of APOE in NSCs during development would be very useful.

 

Other comments/suggestions:

- Sox2 (neural stem and progenitor marker) and Tbr2 (neuronal progenitor marker) stainings are presented only at one stage each. It would be very informative to see if/how these two markers change over the course of the differentiation protocol. If not possible by immuno, a QPCR for these genes would also be enough to show the trends of expression during differentiation.
- Please specify in the methods how long the cells are fixed in PFA.
- Either in the introduction or the discussion, it could be noted that astrocytes express very high levels of APOE in the brain. This is important because it links APOE expression to the astroglial nature of adult NSCs.

Is the work clearly and accurately presented and does it cite the current literature?

Yes

If applicable, is the statistical analysis and its interpretation appropriate?

Yes

Are all the source data underlying the results available to ensure full reproducibility?

Yes

Is the study design appropriate and is the work technically sound?

Yes

Are the conclusions drawn adequately supported by the results?

Partly

Are sufficient details of methods and analysis provided to allow replication by others?

Yes

Reviewer Expertise:

Adult neurogenesis

I confirm that I have read this submission and believe that I have an appropriate level of expertise to confirm that it is of an acceptable scientific standard, however I have significant reservations, as outlined above.

# Response

**Published:** 2020-08-19

**Authors:** Sandrine Thuret

**Affiliations:** King's College London, UK

<>

*Comment 1. If possible, provide higher magnification/higher quality images of APOE stainings, including also the other time points during differentiation. Day 7 would be particularly important to include, since it displays the highest levels of expression by QPCR.*

The authors would like to thank the reviewer for this comment. We agree that the resolution of the images in Figure 2 is not as high as the original data when the manuscript is viewed/downloaded online. **In the updated manuscript, these images are shown in a separate figure (Figure 3).** The PDF version should enable sufficient magnification to view the composite panels and clearly demonstrate more intracellular localisation on D18/19 cells. **In addition, as indicated in the ‘Data availability’ section of the submitted manuscript, the original data with higher resolutions for all images can be found on Figshare, a public repository for scientific data, which can be accessed via the following DOI:** https://doi.org/10.6084/m9.figshare.12199745.v1 **.** We are confident that the patterns of APOE immunocytochemistry (ICC) reported in the manuscript can be confirmed with greater detail from the raw data available on Figshare.

**While the authors confirm that the ICC experiments were conducted for APOE on D7 cells, the data were not included in the manuscript due to the following reasons.** According to the differentiation protocol, the cells were maintained at high density approaching near 100% confluence from D0 to D7. We observed that this inadvertently diminishes the quality of immunocytochemistry images for D7 cells, since clear boundaries of nuclei could not be easily identified with epifluorescence microscopy and further complicated the downstream quantification process. The possibility of dissociating D7 cells and plating them on to a different surface for better image quality and quantification was considered briefly. However, such additional handling was not done to the cells so that any potential source of artefacts that could mask the true state of D7 cells can be ruled out in our experiments. While the use of epifluorescence microscopy in our study can be seen as a clear limitation, APOE immunostaining patterns of D7 cells was not qualitatively different from that of D12 cells in our observations. Further investigations using three-dimensional imaging techniques such as confocal microscopy will enable better imaging and quantification of densely packed cells on D7. **The ‘Discussion’ section in the updated manuscript now includes a new paragraph regarding this aspect.**

“One limitation of this study is that the time-dependent changes of differentiation markers such as SOX2 and TBR2 were not examined alongside APOE. It is worth noting, however, that TBR2 was shown to be capable of suppressing SOX2 expression during differentiation of NSCs to NPCs ( Hodge et al., 2012). Given this information, it is unlikely that TBR2-positive cells observed in this study at D18/19 will simultaneously express high levels of SOX2. However, time-dependent changes of various markers of differentiation would add further validity to our observations and unequivocally clarify whether APOE expression is indeed correlated with the differentiation state of the cells. **Another limitation of this study is that the exact locus of APOE expression could not be examined in detail using a standard epifluorescence microscope in this study. High-resolution microscopy techniques (such as confocal microscopy) would have been more ideal to identify the accurate loci of APOE expression and overcome the challenges of imaging densely packed cells at the earliest stages of neural induction (D0-D7). Further investigations with improved imaging capacity will therefore allow us to characterise APOE during the earlier stages of neural induction and hint at potential mechanisms underlying its role in neurodevelopment.**”

**We do not have the data for other time points during differentiation yet, and we agree with the reviewer that it would be highly informative to examine more time points.** It would be particularly interesting to examine the time-course of APOE expression changes from the stem cell stage to the mature neuronal/glial stage. We hope that the data reported in our manuscript can serve as a foundation to such experiments to be conducted in the future.

*Comment 2. APOE stainings should be quantified to support the conclusions that its expression decreases during differentiation and there is a change in the protein localization. If this is not possible, the conclusions should be toned down and further experiments suggested in the discussion (for example, protein quantification by WB and cellular fractionation and quantification of protein in the media to assess intracellular protein localization and secretion, respectively).*

The authors would like to thank the reviewer for the comment on the quantification of ICC images. We now include a quantification of the images in the updated manuscript. The percentage of SOX2+ cells and TBR2+ cells at D12 and D18/19, as well as the percentage of SOX2+ and TBR2+ cells that showed immunostaining patterns for intracellular APOE were quantified using CellProfiler v3.1.9. **The quantified data presented as bar graphs can be found in Figure 3; the analysis procedure can be found in the ‘Methods (Immunocytochemistry)’ section; and the analysis pipeline as well as the raw data for quantification can be found on Figshare which can be accessed by the following DOI:** https://doi.org/10.6084/m9.figshare.12781064.v1 **.** The results indicate a higher percentage of intracellular APOE/TBR2+ cells at D18/19 compared to intracellular APOE/SOX2+ cells at D12, supporting our conclusions made in the manuscript prior to the inclusion of these quantification data.

To incorporate the reviewer’s suggestions, **we have clearly stated in the ‘Discussion’ section of our updated manuscript that further investigations using other techniques should be done to quantify the levels of APOE protein.** The following changes have been made in the ‘Discussion’.

“In this study, qualitative analysis was performed on APOE immunocytochemistry results. As the cells became more differentiated from NSCs to NPCs, APOE localisation pattern became more intracellular. **To validate this observation, however, additional experiments with a more direct quantitative approach should be conducted. For example, APOE protein levels in various subcellular compartments could be measured and compared by performing Western Blot.** Since APOE has been shown to exist in both secreted and intracellular forms ( Huang & Mahley, 2014), it will be interesting to see which form of APOE is produced at each differentiation stage. It is possible that more APOE is secreted in undifferentiated cells compared to differentiated cells, which may not be fully captured using immunocytochemistry techniques performed on fixed cells. Interestingly, Gan and colleagues previously reported that APOE is indeed secreted by NSCs as well as NPCs, and secreted APOE was found to play a vital role in regulating NSC survival and neurosphere formation ( Gan et al., 2011). **Therefore, further investigations on secreted and intracellular APOE using quantitative approaches will be able to clarify whether cells indeed produce different forms and levels of APOE depending on its differentiation state.** This will in turn provide more definitive clues to whether APOE plays a stage-dependent role in neurodevelopment.”

Furthermore, **the conclusions have been amended as follows to reflect the changes in the ‘Discussion’.**

“In conclusion, we report that human APOE gene expression levels are highly correlated with the undifferentiated state of cells during directed differentiation in vitro, and **ApoE protein is localised more in the intracellular region in cells at later stages of differentiation**. Combining our observations and previous evidence reported in the literature, we speculate that APOE has an important role in stem cell maintenance **and propose that further investigations should be carried out to validate our findings including methods that were not employed in this study**. Moreover, it would be interesting to examine the exact underlying mechanisms such as 1) whether APOE is an upstream or downstream factor of stem cell maintenance, and 2) whether APOE4 genotype and APOE loss-of-function would produce similar phenotypes.”

*Comment 3. In the discussion, it should be noted that iPSC-derived NSCs might not fully resemble adult NSCs. A brief discussion of what is known about the expression of APOE in NSCs during development would be very useful.*

The authors would like to thank the reviewer for this comment on highlighting the differences between iPSC-derived NSCs and adult NSCs. To incorporate this aspect into our updated manuscript, **we have now added a new paragraph in the ‘Discussion’ section as follows**.

“Since NSCs derived from iPSCs in vitro may not fully resemble the developmental and postnatal NSCs found in vivo, APOE expression should be further investigated in animal models of brain development as well. The most direct evidence of in vivo APOE expression in NSCs to this date comes from a study by Yang and colleagues, where Nestin-positive NSCs in the mouse developing dentate gyrus was isolated using fluorescence-activated cell sorting, and APOE expression was examined from as early as postnatal day 7 (P7) ( Yang et al., 2011). NSCs at P7 had low expression of APOE which increased with the age of mice, and the deletion of APOE had detrimental effects on the maintenance of stem cells in the dentate gyrus. Although these findings clearly demonstrate the importance of APOE in brain development, the study had limitations in that prenatal NSCs were not examined, and functional studies of APOE were based on global rather than conditional knockouts. Furthermore, Yang and colleagues’ data cannot be directly compared with our dataset due to species difference and the lack of detailed characterisation of NSCs in this study. To address this knowledge gap, more data from both in vitro and in vivo samples derived from various species should be generated and compared against each other. We hope that our focused study has laid a strong foundation to such collaborative investigations that may be conducted in the future.”

<>

*Comment 1. Sox2 (neural stem and progenitor marker) and Tbr2 (neuronal progenitor marker) stainings are presented only at one stage each. It would be very informative to see if/how these two markers change over the course of the differentiation protocol. If not possible by immuno, a QPCR for these genes would also be enough to show the trends of expression during differentiation.*

The authors would like to thank the reviewer for pointing out the expression changes of SOX2 and TBR2. While we have not examined the time-dependent changes of these markers in this study, the authors can confirm that SOX2 and TBR2 expression was consistently observed at D12 and D18/19 by other experienced members of the lab using the differentiation protocols reported in this study. These data (although not shown in this study) were generated by qPCR, ICC, and microarray experiments that collectively show the expression of SOX2 and TBR2 similar to the ICC data reported in our manuscript. While we are confident with the SOX2 and TBR2 expression pattern in our study, we agree with the reviewer that APOE expression should be examined alongside the differentiation markers. To incorporate this into our updated manuscript, **we have now included a new paragraph in the ‘Discussion’ section** as follows to suggest that further investigations should be conducted to address this aspect.

“ **One limitation of this study is that the time-dependent changes of differentiation markers such as SOX2 and TBR2 were not examined alongside APOE. It is worth noting, however, that TBR2 was shown to be capable of suppressing SOX2 expression during differentiation of NSCs to NPCs ( Hodge et al., 2012). Given this information, it is unlikely that TBR2-positive cells observed in this study at D18/19 will simultaneously express high levels of SOX2. However, time-dependent changes of various markers of differentiation would add further validity to our observations and unequivocally clarify whether APOE expression is indeed correlated with the differentiation state of the cells.** Another limitation of this study is that the exact locus of APOE expression could not be examined in detail using a standard epifluorescence microscope in this study. High-resolution microscopy techniques (such as confocal microscopy) would have been more ideal to identify the accurate loci of APOE expression and overcome the challenges of imaging densely packed cells at the earliest stages of neural induction (D0-D7). Further investigations with improved imaging capacity will therefore allow us to characterise APOE during the earlier stages of neural induction and hint at potential mechanisms underlying its role in neurodevelopment.”

*Comment 2. Please specify in the methods how long the cells are fixed in PFA.*

**The authors can confirm that the cells were fixed for 10 mins at room temperature with 4% PFA, and this has been now clearly stated in the ‘Methods’ section.** We thank the reviewer for pointing this out.

“ **Cells were fixed with 4% paraformaldehyde for 10 mins at room temperature**, permeabilized with 0.1% Triton™ X-100 in 1X Tris-buffered saline (TBS) for 15–30 minutes, and then blocked with 5% normal donkey serum in TBS for 30 minutes. Primary antibodies were incubated at 4°C overnight followed by 3 washings with TBS. Secondary antibodies conjugated with fluorescent dyes were incubated at room temperature for 1 hours followed by 2 washings with TBS. Nuclei were stained with 5 µg/mL Hoechst® 33342 solution (Thermo Fisher) for 30 seconds immediately prior to imaging, and cells were washed with TBS 2 times after nuclear staining. All primary antibodies were diluted in 5% normal donkey serum in TBS, secondary antibodies in 1% normal donkey serum in TBS, and Hoechst® 33342 solution in TBS. Imaging was done with IX 70 inverted epifluorescence microscope (Olympus) connected to AxioVision imaging software (version 4.4). Scale bars were inserted on the images using ImageJ software (version 1.49c). CellProfiler (version 3.1.9) was used to quantify the percentage of cells immunopositive for SOX2, TBR2, and APOE at the intracellular regions. Raw dataset for the images is available as an underlying data via Figshare (doi: 10.6084/m9.figshare.12199745.v1). Raw dataset for the quantification is available as an underlying data via Figshare (doi: 10.6084/m9.figshare.12781604.v1).”

*Comment 3. Either in the introduction or the discussion, it could be noted that astrocytes express very high levels of APOE in the brain. This is important because it links APOE expression to the astroglial nature of adult NSCs.*

The authors would like to thank the reviewer’s comment on the existing evidence of APOE expression in astrocytes. To incorporate this aspect into our updated manuscript **, the ‘Introduction’ section has been amended as follows**.

“ **Apolipoprotein E (APOE) is a pleiotropic protein that plays an important role in lipid metabolism ( Mahley & Rall, 2000) and is highly expressed in the brain mainly by glial cells ( Elshourbagy et al., 1985, Boyles et al., 1985).** Although the primary function of APOE is lipid transport, its expression is also found in other cell types outside the context of lipid metabolism in the brain ( Liao et al., 2017). For example, a recent single-cell RNA sequencing study on human post-mortem Alzheimer’s disease (AD) brains showed that activated microglia (relevant to the disease state) express high levels of APOE unlike naïve microglia (relevant to healthy/homeostatic state) in the prefrontal cortex, indicating that APOE expression is associated with immune function ( Mathys et al., 2019). Furthermore, neuronal APOE can also be expressed at high levels under stress conditions such as brain injury although APOE expression is normally low in healthy neurons ( Mahley & Huang, 2012; Xu et al., 2006). Interestingly, APOE is highly expressed in nestin/glial fibrillary acidic protein (GFAP) double-positive neural stem cells (NSCs) in the adult hippocampus of mice, and one of the phenotypes characterised in APOE-null mice is the premature depletion of NSC pool in the hippocampus, suggesting that NSC maintenance requires APOE expression ( Yang et al., 2011).”

# Peer review: Reviewer response for version 1

**DOI:** 10.5256/f1000research.26021.r63366

**Published:** 2020-06-08

**Authors:** Robert J. Williams

**Affiliations:** 1 Department of Biology and Biochemistry, University of Bath, Bath, UK

**Summary:**

 It has previously been shown that APOE regulates neural stem cell (NSC) maintenance in rodent models but until now this has not been demonstrated or validated in human cells. The authors address this important question through use of human induced pluripotent stem cells (APOE3 homozygous) and monitor APOE status throughout neural differentiation. APOE gene expression was notably much lower following induction of differentiation and coincident with this was an apparent change in the cellular distribution of APOE with the authors describing a much more pronounced intracellular localisation of APOE. 

**Major comments:**

 This is a very focused piece of work and although the findings are quite preliminary they are very interesting and worth reporting particularly because of the human cell context. A key question going forward will be whether the APOE4 genotype influences NSC maintenance or differentiation as this could have clear implications for neurogenesis in Alzheimer’s Disease. The gene expression data presented here is clear and the changes in APOE are quite dramatic but the immunostaining is rather descriptive and is not quite as convincing.

**Specific comments:**

1. The resolution of the images in the composite cell panels in Fig 2 is not great and it is difficult to clearly see or judge the intracellular expression described. Can these be improved at all?
2. For the gene expression changes, data is provided for D7, D12 and D18, yet for the cell staining only D12 and D18 are shown. Is D7 cell data not available to present here?
3. The most obvious change at the protein level is the apparent increase in cellular APOE staining between D12 and D18 (certainly for DSi and DS-Wi) and as Hoe33342 has been performed I would have thought some quantitative data could be provided here which would help support the conclusions made.

Is the work clearly and accurately presented and does it cite the current literature?

Yes

If applicable, is the statistical analysis and its interpretation appropriate?

Yes

Are all the source data underlying the results available to ensure full reproducibility?

Yes

Is the study design appropriate and is the work technically sound?

Yes

Are the conclusions drawn adequately supported by the results?

Partly

Are sufficient details of methods and analysis provided to allow replication by others?

Yes

Reviewer Expertise:

Molecular and Cellular Neuroscience

I confirm that I have read this submission and believe that I have an appropriate level of expertise to confirm that it is of an acceptable scientific standard.

# Response

**Published:** 2020-08-19

**Authors:** Sandrine Thuret

**Affiliations:** King's College London, UK

*Comment 1. The resolution of the images in the composite cell panels in Fig 2 is not great and it is difficult to clearly see or judge the intracellular expression described. Can these be improved at all?*

The authors would like to thank the reviewer for this comment. We agree that the resolution of the images in Figure 2 is not as high as the original data when the manuscript is viewed/downloaded online. **In the updated manuscript, these images are shown in a separate figure (Figure 3).** The PDF version should enable sufficient magnification to view the composite panels and clearly demonstrate more intracellular localisation on D18/19 cells. **In addition, as indicated in the ‘Data availability’ section of the submitted manuscript, the original data with higher resolutions for all images can be found on Figshare, a public repository for scientific data, which can be accessed via the following DOI:** https://doi.org/10.6084/m9.figshare.12199745.v1 **.** We are confident that the patterns of APOE immunocytochemistry (ICC) reported in the manuscript can be confirmed with greater detail from the raw data available on Figshare.

*Comment 2. For the gene expression changes, data is provided for D7, D12 and D18, yet for the cell staining only D12 and D18 are shown. Is D7 cell data not available to present here?*

The authors would like to thank the reviewer for mentioning this important aspect of the ICC experiment reported in our manuscript. **While the authors confirm that the ICC experiments were conducted for APOE on D7 cells, the data were not included in the manuscript due to the following reasons.** According to the differentiation protocol, the cells were maintained at high density approaching near 100% confluence from D0 to D7. We observed that this inadvertently diminishes the quality of immunocytochemistry images for D7 cells, since clear boundaries of nuclei could not be easily identified with epifluorescence microscopy and further complicated the downstream quantification process. The possibility of dissociating D7 cells and plating them on to a different surface for better image quality and quantification was considered briefly. However, such additional handling was not done to the cells so that any potential source of artefacts that could mask the true state of D7 cells can be ruled out in our experiments. While the use of epifluorescence microscopy in our study can be seen as a clear limitation, APOE immunostaining patterns of D7 cells was not qualitatively different from that of D12 cells in our observations. Further investigations using three-dimensional imaging techniques such as confocal microscopy will enable better imaging and quantification of densely packed cells on D7. **The ‘Discussion’ section in the updated manuscript now includes a new paragraph regarding this aspect.**

“One limitation of this study is that the time-dependent changes of differentiation markers such as SOX2 and TBR2 were not examined alongside APOE. It is worth noting, however, that TBR2 was shown to be capable of suppressing SOX2 expression during differentiation of NSCs to NPCs ( Hodge et al., 2012). Given this information, it is unlikely that TBR2-positive cells observed in this study at D18/19 will simultaneously express high levels of SOX2. However, time-dependent changes of various markers of differentiation would add further validity to our observations and unequivocally clarify whether APOE expression is indeed correlated with the differentiation state of the cells. **Another limitation of this study is that the exact locus of APOE expression could not be examined in detail using a standard epifluorescence microscope in this study. High-resolution microscopy techniques (such as confocal microscopy) would have been more ideal to identify the accurate loci of APOE expression and overcome the challenges of imaging densely packed cells at the earliest stages of neural induction (D0-D7). Further investigations with improved imaging capacity will therefore allow us to characterise APOE during the earlier stages of neural induction and hint at potential mechanisms underlying its role in neurodevelopment.**”

*Comment 3. The most obvious change at the protein level is the apparent increase in cellular APOE staining between D12 and D18 (certainly for DSi and DS-Wi) and as Hoe33342 has been performed I would have thought some quantitative data could be provided here which would help support the conclusions made.*

The authors would like to thank the reviewer for the comment on the quantification of ICC images. We now include a quantification of the images in the updated manuscript. The percentage of SOX2+ cells and TBR2+ cells at D12 and D18/19, as well as the percentage of SOX2+ and TBR2+ cells that showed immunostaining patterns for intracellular APOE were quantified using CellProfiler v3.1.9. **The quantified data presented as bar graphs can be found in Figure 3; the analysis procedure can be found in the ‘Methods (Immunocytochemistry)’ section; and the analysis pipeline as well as the raw data for quantification can be found on Figshare which can be accessed by the following DOI:** https://doi.org/10.6084/m9.figshare.12781064.v1 **.** The results indicate a higher percentage of intracellular APOE/TBR2+ cells at D18/19 compared to intracellular APOE/SOX2+ cells at D12, supporting our conclusions made in the manuscript prior to the inclusion of these quantification data.
